# Recombinant humanised anti-HER2/neu antibody (Herceptin®) induces cellular death of glioblastomas

**DOI:** 10.1038/sj.bjc.6602089

**Published:** 2004-08-24

**Authors:** J-F Mineo, A Bordron, I Quintin-Roué, S Loisel, K L Ster, V Buhé, N Lagarde, C Berthou

**Affiliations:** 1 Department of Neurosurgery, University Medical School Hospital of Brest, BP 824, F29609 Brest Cedex, France; 2 Laboratory of Hematology, University Medical School Hospital of Brest, BP 824, F29609 Brest Cedex, France; 3 Laboratory of Pathoanatomy, University Medical School Hospital of Brest, BP 824, F29609 Brest Cedex, France

**Keywords:** glioblastoma, monoclonal antibody, HER2/neu, apoptosis, cytotoxicity

## Abstract

Glioblastoma multiforme (GBM) remains the most devastating primary tumour in neuro-oncology. Targeting of the human epithelial receptor type 2 (HER2)-neu receptor by specific antibodies is a recent well-established therapy for breast tumours. Human epithelial receptor type 2/neu is a transmembrane tyrosine/kinase receptor that appears to be important for the regulation of cancer growth. Human epithelial receptor type 2/neu is not expressed in the adult central nervous system, but its expression increases with the degree of astrocytoma anaplasia. The specificity of HER2/neu for tumoral astrocytomas leads us to study *in vitro* treatment of GBM with anti-HER2/neu antibody. We used human GBM cell lines expressing HER2/neu (A172 express HER2/neu more than U251MG) or not (U87MG) and monoclonal humanised antibody against HER2/neu (Herceptin®). Human epithelial receptor type 2/neu expression was measured by immunohistochemistry and flow cytometry. Direct antibody effect, complement-dependent cytotoxicity and antibody-dependent cellular cytotoxicity were evaluated by different cytometric assays. We have shown, for the first time, the ability of anti-HER2/neu antibodies to induce apoptosis and cellular-dependent cytotoxicity of HER2/neu-expressing GBM cell lines. The results decreased from A172 to U251 and were negative for U87MG, in accordance with the decreasing density of HER2/neu receptors.

Glioblastomas multiforme (GBM) are the most common malignant tumours of the central nervous system. With an incidence from 0.4 to 2.8 per year per 100 000 persons (De Triboulet, 1996; Mineo *et al*, 2002), they are ranked fourth among the causes of death due to cancer in the middle-aged man (Rainov *et al*, 1997).

Glioblastoma multiforme are partially refractory to radiation and chemotherapy that are in standard use today (medial survival of 18 months despite radical surgery; Hiesiger *et al*, 1993; Puzzili *et al*, 1998). The poor effectiveness of current therapy evokes development in areas, used for treatment of other cancers, such as immunotherapy. Human monoclonal antibody treatment against human epithelial receptor type 2 (HER2)/neu overexpressing breast cancer increases patient survival (Baselga *et al*, 1996). The effect of this antibody has led oncologists to prescribe immunotherapy in metastatic breast cancer. The HER2/neu is a 185 kDa transmembrane tyrosine/kinase receptor, which is a member of the tyrosine kinase receptor family. It is involved in the regulation of cell growth and in differentiation, especially during brain embryogenesis. The HER2/neu (also called c-erbB2) receptor is not found in the adult central nervous system (Press *et al*, 1990), but it appears in tumoral astrocytes. Its expression increases with the degree of astrocytoma anaplasia (Kristt and Yarden, 1996) and becomes frequent in glioblastomas (20–90%; Bian *et al*, 2000; Forseen *et al*, 2002; Koka *et al*, 2003). Overexpression of HER2/neu seems correlated to higher degree of glioma cells anaplasia (Kristt and Yarden, 1996). Activated HER2/neu can form homologous complexes, but the major action of HER2/neu results from heterodimerisation of HER2/neu with the other tyrosine kinase family-activated receptors (like epidermal growth factor receptor also called c-erbB1; Harwerth *et al*, 1993). Ligand–receptor complexes that include HER2/neu appear to be more potent that other receptor complexes and have a higher ligand affinity, a lower rate of internalisation and degradation and a higher tyrosine kinase activity (Sliwkowski *et al*, 1999). Human epithelial receptor type 2/neu inhibition could therefore not only decrease the activity of HER2/neu but also could affect the activity of other tyrosine kinase receptors. Owing to the association of HER2/neu proto-oncogene overexpression and human cancer, some monoclonal antibodies against a panel of epitopes from HER2/neu receptor were developed in animals. A more potent antibody for tumour inhibition was fully humanised for human therapeutic administration to create trastuzumab (Herceptin®). The targeting of HER2/neu by its specific antibody is known to have antitumoral effect in breast cancer (Pietras *et al*, 1998; Spiridon *et al*, 2002). The activated antibody containing human Fc region can generate two cytolytic pathways against the target cells by the activation of the complement cascade and antibody-dependent cellular cytotoxicity (ADCC). The ADCC is triggered by interaction between antibody-coated target cells and Fc receptor type III (CD16) on effector cells (Carter *et al*, 1992), and as resting microglia cells are found to express constitutively Fc receptors, local ADCC (Vedeler *et al*, 1994) could be induced by trastuzumab in the brain. Moreover, intravital fluorescence microscopical approach demonstrates that glioma microvascularisation produces an abnormal blood–brain barrier. The intravital approach (Vajkoczy *et al*, 1998) shows an intratumoral homogeneous extravasation of a 150-kDa protein (weight of IgG).

In our study, Herceptin® induces GBM cell lines apoptosis and ADCC. These effects are correlated to HER2/neu expression. These results suggest that HER2/neu might be a useful tumour-specific target for antibody-mediated therapy.

## MATERIALS AND METHODS

### Cells lines

The GBM cell lines were grown in Dulbecco's modified Eagle's medium (Gibco, Paisley, Scotland) supplemented with 10% heat-inactivated foetal calf serum (Gibco, Paisley, Scotland), 1% L-glutamine (Eurobio), 100 UI ml^−1^ penicillin G and 100 *μ*g ml^−1^ streptomycin (Eurobio) and maintained at 37°C in a humid atmosphere of 5% CO_2_ in air. Before the experiments, cells were harvested by 0.05% trypsin and 0.02% EDTA (Eurobio) at 37°C; their viability was more than 95% as assessed by the trypan blue technique. The U87MG, A172 and U251MG human glioblastoma cell lines were kindly provided, respectively, by F Furnari (Ludwig Institute For Cancer Research, La Jolla, CA, USA), JL Fischel (Centre A Lacassagne, Nice, France) and A Gatignol (Lady Davis Institute for Medical Research, Montreal, Quebec, Canada).

### Monoclonal antibody

Herceptin® (trastuzumab, Genentech, Roche Laboratory) is a humanised monoclonal immunoglobulin G against HER2/neu extracellular epitope (amino acids 529–627). It derivates from a murine antibody (called 4D5 (Fendly *et al*, 1990; Lewis *et al*, 1993) and it was humanised for clinical administration. The hypervariable domain remains similar to the animal domain, but the other 95% is similar to human IgG1. For experimental use, Herceptin®, in lyophilised form, was weighted daily, and suspended in phosphate-buffered saline (PBS).

### Immunohistochemistry

Sections of cellular pellet were exposed to hydrogen peroxide to reduce endogenous peroxidase activity and then exposed to pig serum to block the nonspecific protein binding. The sections were incubated with a rabbit anti-human polyclonal antibody against HER2/neu oncoprotein (Dakocytomation, Denmark) diluted 1 : 250 in PBS 0.1 M 30 min at room temperature. After three rinses in the same buffer, the sections were incubated with a polymer conjugated with peroxidase and anti-rabbit immunoglobulins. Colour was developed by incubation with diaminobenzidine and H_2_O_2_. Finally, the sections were washed in PBS, dehydrated and coverslips were added for optical microscopy.

### Flow cytometry analysis

A total of 2 × 10^5^ cells were labelled for 30 min at 4°C with 10 *μ*g of Herceptin®, washed twice by PBS supplemented with 1% bovine serum albumin (PBS-BSA), then incubated with fluorescein isothiocyanate (FITC)-conjugated rabbit anti-human IgG (1 : 10 dilution, 30 min at 4°C) (Dakocytomation, Denmark). Two washes were carried out again before cytometric analysis.

The complement inhibitors, CD55 (decay accelerating factor), CD59 (Protectin) and CD45 (complement receptor type 1) were detected at the cell surface as in the previous experiment. We used the following antibodies: FITC-labelled mouse anti-human CD55 (Beckman Coulter), FITC-conjugated mouse anti-human CD45 (Beckman Coulter) and unconjugated mouse anti-human CD59 (Beckman Coulter) revelled by a phycoerythrin (PE)-conjugated goat anti-mouse IgG. Per experiment, 5000 cells were analysed in a flow cytometer (Beckman Coulter) equipped with a 500 mW argon laser. The flow cytometer settings selected were controlled by using unstained cells and isotype-matched nonreactive FITC Mabs. The number of fluorescent molecules per cell were indirectly measured by assessing the mean fluorescence intensity (MFI) of cells analysed in each test.

### Identification of apoptotic cells

The phosphatidyl serine (PS) translocation to the outer face of the membrane was visualised by the binding of Annexin V. A total of 105 cells in 1 ml culture medium were treated with 1 ml of different concentrations of Herceptin®. After 6, 12 or 24 h incubation at 37°C, cells were washed in PBS and stained with FITC–Annexin V and propidium iodide (PI) according to the manufacturer's instructions (Beckman Coulter). PI was used to exclude dead cells. Percentages of apoptotic cells (Annexin V-positive cells) were thus calculated within the PI-negative population of cells. To analyse the different data, some results were expressed as the percentage of variation which is equal to


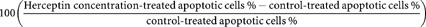


### Complement-dependent cytotoxicity assay

To study the effect of Herceptin® in inducing the activation of complement, 10^5^ cells were incubated for 6, 12 or 24 h with different Herceptin® concentrations containing 15% of AB human serum (used as source of complement and given by volunteers and consent donors of the Transfusion Center, Brest). The cells were removed and PI was added for 10 min at 4°C before cytometric analysis.

### Antibody-dependent cellular cytotoxicity assay

The peripheral large granular lymphocytes (LGL, including natural killer lymphocytes), used as human effector cells, were separated from the blood of healthy donors in density gradients (first on Ficoll–Hypaque density gradients and then on Percoll (Pharmacia) density gradients). A total of 2 × 104 GBM cells (targets) and LGL (effectors) were mixed in 1 ml culture medium (ratio targets/effectors 1/75 or 1/100) and then incubated for 6 h at 37°C with 1 ml of antibodies. To stain effectors, we incubated the washed cells with 5 *μ*g of FITC-conjugated anti-human CD45 antibodies for 30 min at room temperature (CD45 is not present on glioblastomas cells). Two further rinses in PBS were carried out before cytometric analysis, cells were then incubated for 10 min at 4°C with 10 *μ*g of PI to determine the percentages of dead cells.

### Statistical analysis

The data were analysed for significance by Student's *t*-test.

## RESULTS

### GBM cell lines express HER2/neu

Human epithelial receptor type 2/neu membranous density was evaluated by immunohistochemistry (IHC) and flow cytometry. Histopathologists classified immunostaining as follows (as used for breast cancer): 0=no staining; 1+=faint, incomplete membranous pattern; 2+=moderate, complete membranous pattern; and 3+=strong membranous pattern (Forseen *et al*, 2002). For the cytometric assay, the density was estimated by comparative method between the MFI of isotype control and the MFI after HER2/neu-FITC staining. BT474 is an overexpressing 3+ breast cancer cell line used as reference in most studies. Its MFI was 20. By cytometry, the MFI of A172 cell line was 4; this cell line was classified + by HIC. The MFI of U251 cell line was 2, indicating a less important intensity of HER2/neu molecule per cell than for A172; U251MG was classified 0 by HIC. Based on these two different techniques, we noted that U87MG cell line showed no membranous HER2/neu positivity (Table 1

**Table 1:** Evaluation of HER2/neu expression on glioblastoma cell lines by IHC and by flow cytometry

		**HER2/neu expression**	
**Cell lines**	**Species**	**IHC**	**Flow cytometry**
GL15	Human	−	−
U87	Human	−	−
8MG	Human	−	Weak
LN18	Human	−	Weak
T98G	Human	−	Weak
U251MG	Human	−	+
A172	Human	+	++
BT474	Human/breast	+++	+++

HER2=human epithelial receptor type 2; IHC=immunohistochemistry.

). The effect of Herceptin® was tested on the U251MG and A172 cell lines. The U87MG cell line was used as a negative cell line for the expression of HER2/neu.

### Anti-HER2/neu antibody induces glioblastomas cells apoptosis

**Figure 1:**
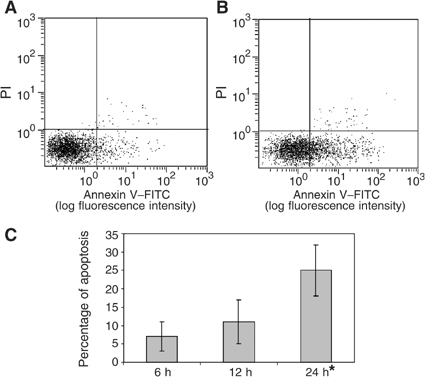
Apoptosis induction: The A172 cell line expressing HER2/neu undergo apoptosis, following incubation with Herceptin®. Cells were incubated for 24 h with medium (**A**) or with 25 *μ*g of Herceptin® (**B**). The cells staining with FITC-conjugated Annexin V and PI. Apoptotic cells were identified as Annexin V positive and PI negative. (**C**) Time course analysis of PS exposure after incubation of A172 cell line Herceptin® 25 *μ*g ml^−1^. Only 24 h results are statistically significant (*P*<0.01).

**Figure 2:**
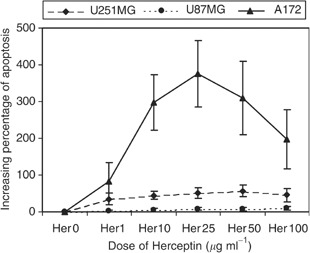
Increasing apoptosis in glioblastomas cell lines expressing HER2/neu: Cells were incubated for 24 h with different concentrations of Herceptin®.

Herceptin® induces the apoptosis of GBM cell lines expressing HER2/neu, as shown by staining by Annexin V of A172 cells incubated with anti-HER2/neu antibody and nearly no staining by PI (Figure 1

). Best results were obtained at 24-h cell incubation with antibodies (6 h tests showed less activity and 48 h tests did not show higher apoptosis; Figure 1C). When mixed with A172 cell line, the efficiency of the antibody increased with the antibody concentration until 25 *μ*g ml^−1^ and then decreased (probably corresponding to the saturation of antibody recognition sites). The spontaneous apoptosis without antibody of 10 tests were compared by Student's *t*-test to the apoptosis with 25 *μ*g ml^−1^ of antibody, the test was significant with *P*<0.01. With U251MG cell line, the highest activity was observed with 50 *μ*g ml^−1^l (comparison between the apoptosis without or with 50 *μ*g ml^−1^ of antibody of five tests was significant with *P*<0.05, Figure 2

). Moreover, the higher apoptosis induction was obtained in the A172 cell line than in the U251MG cell line. No apoptosis induction was found for U87MG cell line. The apoptosis induction decreases in accordance with the density of HER2/neu receptors.

### Complement-dependent cytotoxicity assay

We wanted to evaluate the ability of Herceptin® to induce complement-mediated cytotoxicity on HER2/neu-expressing GBM cell lines. For this purpose, human serum AB (used as source of complement) was added to the medium with antibodies, but it did not increase cell death induction. The inefficiency of complement-mediated cytotoxicity was explained by a strong expression of complement inhibitory factors (CD55 and CD59 on A172 and U251MG cell lines, data not shown).

### ADCC of anti-HER2/neu antibody

**Figure 3:**
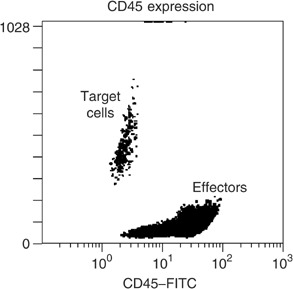
CD45 expression on the GBM cell lines and leucocytes: Cells were stained by FITC-conjugated mouse anti-human CD45 for 30 min at 4°C. Flow cytometry allowed us to differentiate the glioblastoma and effector cells.

**Figure 4:**
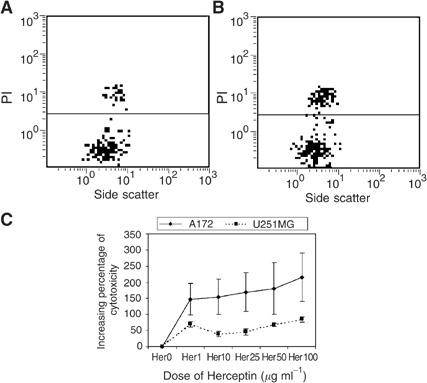
Antibody-dependent cellular cytotoxicity of Herceptin® against A172 cell line. Target cells were incubated with effector cells (ratio targets/effectors 1/75) for 6 h with medium only (**A**) or with 10 *μ*g ml^−1^ of Herceptin® (**B**). FITC labelled anti-human CD45 antibody then PI was added. Analysis was based on the negative A172 population CD45 determination. The dead cells were stained by PI (**C**).

During ADCC assay establishment, we measured the density of membranous CD45. We observed that this molecule was highly expressed by effector cytotoxic cells. This allowed us to differentiate the two cellular types (GBM and effectors; Figure 3

) and to analyse ADCC (Figure 4

). In order to determine the level of cytotoxicity induced by the antibody and by the effectors alone, control tests were performed using Herceptin® alone without effectors and effectors alone without antibody. Herceptin® did not induce apoptosis in U87MG cell line and ADCC was not tested for this cell line. During this assay, we observed that ADCC was already presented using 1 *μ*g ml^−1^ of antibody (comparison between the cellular death without or with 1 *μ*g ml^−1^ of antibody of five tests was significant with *P*<0.05) and was still moderately increased with higher concentrations (Figure 4C). Moreover, as apoptosis was mediated by Herceptin®, more cytotoxicity was induced in A172 cell line than in U251MG cell line. Similar results were obtained with target/effector ratios of 1/75 and 1/100.

## DISCUSSION

This study demonstrates, for the first time, the ability of Herceptin® to induce *in vitro* apoptosis of HER2/neu-expressing GBM.

Of the cases studied (results from tumour biopsy), the range in results observed for HER2/neu *in vivo* positivity is wide (20–90%). Some authors detect HER2/neu positivity without density analysis of receptors, so they report more than 70% positivity (Dietzmann and Von Bossanyi, 1994; Kristt and Yarden, 1996; Westphal *et al*, 1997; Bian *et al*, 2000). Some authors consider only the 2+ and 3+ tumours as positive, so they report only 20% positivity (Forseen *et al*, 2002; Koka *et al*, 2003).

The low number of cell lines overexpressing HER2/neu *in vitro* was unexpected (more than 70% *in vivo* positivity and only 2 positivity from seven different human cell lines, Table 1). Random selection may explain the difference but it seems that very few cell lines *in vitro* overexpress tyrosine kinase receptors I (EGFR) or II (HER2/neu (Thomas *et al*, 2003). Some authors report decreasing of tyrosine kinase receptor cellular density during long *in vitro* culture and increasing receptor density on *in vivo* implantation (Fischel, 2003). Paracrine stimulation of the receptor by its ligand *in vivo* could explain this difference.

The effect of Herceptin® was first demonstrated for breast cancer (Pegram *et al*, 1999), then apoptotic effects were reported in different cancers (Büchler *et al*, 2001; Fujimura *et al*, 2002; Kono *et al*, 2002). We report similar results with GBM cell lines: Herceptin® induces a significant apoptosis induction at 24 h. Most studies reported significant results after 72 h except one on ovarian adenocarcinoma, which was significant after 24 h (Fujimura *et al*, 2002). Two factors could explain this faster effect on GBM cell lines. First, GBM is a very aggressive tumour with a short doubling time. These particular high kinetics could explain an earlier effect. Second, we used Annexin V–IP assay that could be positive earlier than thymidine incorporation assay often describes in other studies.

We obtained greatest apoptosis induction in the A172 cell line than in U251 cell line and no apoptosis in the U87MG cell line. In the same way, the HER2/neu receptor density decreases from the A172 cell line to negative for U87MG. In our study, the effect of Herceptin® was correlated with the HER2/neu receptors density. This correlation was previously reported for breast, gastric and ovarian tumours (Büchler *et al*, 2001; Fujimura *et al*, 2002).

Moreover, we report less apoptosis or cytotoxicity induction by antibodies with GBM cell lines than in most other publications about Herceptin® in other cancer cell lines. The cell lines we used, however, exhibited less HER2/neu overexpression than most other cell lines usually tested with Herceptin®. The MFI of BT474 breast cancer cell lines was with our technical conditions of 20. The MFI of A172 cell line was 4 and the MFI of U251MG cell line was 2. The ability of Herceptin® to induce apoptosis or cytotoxicity seems to be correlated with HER2/neu receptor density in cancer cell lines from the same organ, so the lower receptor density of our cancer cell lines could explain the lower activity of Herceptin®.

Furthermore, Herceptin® is known to induce ADCC in different organ cancer cell lines (Carter *et al*, 1992; Sliwkowski *et al*, 1999). We report the ability of Herceptin® to induce ADCC with higher cytotoxicity in the A172 cell line than the U251MG cell line. The antibody inducing ADCC is again higher in the cell line with the highest HER2/neu density. This result suggests a correlation between the HER2/neu receptors density and the effect of ADCC. Similar correlation was reported for gastric adenocarcinomas (Pegram *et al*, 1999). Microglia was found to make up 7.5–9% of the total glial population in white matter (Akiyama and Mc Geer, 1990). These cells have a leucocyte origin (Flugel *et al*, 2001), phagocytic function and can produce a cytotoxic action when triggered by antibody coated through Fc gamma receptors (Peress *et al*, 1993; Vedeler *et al*, 1994). Proteins (150 kDa) such as IgG are supposed to cross the abnormal GBM blood–brain barrier (Vajkoczy *et al*, 1998). The ability of IgG against EGFR to cross the blood brain barrier was confirmed by a phase I study (Faillot *et al*, 1996). Herceptin® could interact with microglia to produce ADCC *in vivo*.

Adding human serum did not induce cytotoxicity in our GBM cell lines. However, the ability of Herceptin® to induce cytotoxicity with complement is well established (Niculescu *et al*, 1992). We explain the lack of effect to the overexpression by A172 and U251MG cell lines of the two complement inhibitors: CD55 and CD59.

We have shown the efficacy of Herceptin® to induce apoptosis against relative low HER2/neu-overexpressing GBM cell lines by classical IHC. No induction of apoptosis was observed for breast cell lines with such level of HER2/neu expression. It is known that one of the differences between GBM and other cancers is the absence of expression of HER2/neu on the surface of normal glial tissue. Cells from other normal organ (breast or ovarian) express a weak density of HER2/neu. This difference could explain the greater importance of HER2/neu activity on GBM physiology and the greater effect of its blockage. Moreover, the effect of Herceptin® could be increased by synergistic effects with several chemotherapies (as etoposide or cisplatin; Pegram *et al*, 1999).

Finally, the evidence of Herceptin's ability to induce apoptosis or cytotoxicity in GBM cell lines overexpressing HER2/neu made us commence animal experiments in the following months.

## References

[bib1] Akiyama H, Mc Geer PL (1990) Brain microglia constituvely express beta-2 integrins. J Neuroimmunol 30(1): 81–93 1977769 10.1016/0165-5728(90)90055-r

[bib2] Baselga J, Tripathy D, Mendelsohn J, Baughman S, Benz CC, Dantis L, Sklarin NT, Seidman AD, Hudis CA, Moore J, Twaddell T, Henderson IC, Norton L (1996) Phase II study of weekly intravenous recombinant humanized anti-p185 HER2 monolclonal antibody in patients with HER2/neu-overexpressing metastatic breast cancer. J Clin Oncol 14(3): 737–744 8622019 10.1200/JCO.1996.14.3.737

[bib3] Bian XW, Shi JQ, Liu FX (2000) Pathologic significance of proliferative activity and oncoprotein expression in astrocytic tumors. Anal Quant Cytol Histol 22(6): 429–437 11147296

[bib4] Büchler P, Reber H, Büchler M, Roth M, Büchler M, Friess H, Isacoff W, Hines O (2001) Therapy for pancreatic cancer with a recombinant humanized anti-HER2 antibody. J Gastrointest Surg 5(2): 139–146 11331475 10.1016/s1091-255x(01)80025-1

[bib5] Carter P, Presta L, Gorman CM, Ridway JB, Henner D, Wong WL, Rowland AM, Kotts C, Carver ME, Shepard HM (1992) Humanization of an anti-p185 HER2 antibody for human cancer therapy. Proc Natl Acad Sci USA 89(10): 4285–4289 1350088 10.1073/pnas.89.10.4285 PMC49066

[bib6] De Tribolet N (1996) Tumeurs gliales de l'adulte. In Neurochirurgie Decq et Keravel (ed) pp 110–118, Paris: Edition Ellipse

[bib7] Dietzmann K, Von Bossanyi P (1994) Coexpression of epidermal groth factor receptor protein and c-erbB2 oncoprotein in human astrocytic tumors. Zentralbl Pathol 140(4–5): 335–341 7826981

[bib8] Faillot T, Magdelénat H, Mady E, Stasiecki P, Fohanno D, Gropp P, Poisson M, Delattre JY (1996) A phase I study of an anti-epidermal growth factor receptor monoclonal antibody for the treatment of malignant gliomas. Neurosurgery 39(3): 478–483 8875477 10.1097/00006123-199609000-00009

[bib9] Fendly BM, Winget M, Hudziak RM, Lipari M, Napier MA, Ullrich A (1990) Characterization of murine monoclonal antibodies reactive to either the human growth factor receptor or HER2/neu gene product. Cancer Res 50(1): 1550–1558 1689212

[bib10] Fischel JL (2003) Personal Oral Communication. Nice, France: Centre A Lacassagne

[bib11] Flugel A, Bradl M, Kreutzberg GW, Graeber MB (2001) Transformation of donor-derived bone narrow precursor into host microglia during autoimmune CNS inflammation and during the retrograde response to axotomy. J Neurosci Res 66(1): 74–82 11599003 10.1002/jnr.1198

[bib12] Forseen SE, Potti A, Koka V, Koch M, Fraiman G, Levitt R (2002) Identification and relationship of HER2/neu overexpression to short-term mortality in primary malignant brain tumors. Anticancer Res 22(3): 1599–1602 12168843

[bib13] Fujimura M, Katsumata N, Tsuda H, Uchi N, Miyaka S, Hidaka T, Sakai M, Saito S (2002) HER2 is frequently over-expressed in ovarian clear cell adenocarcinoma: possible novel treatment modality using recombinant monoclonal antibody against HER2. Jpn J Cancer Res 93(11): 1250–1257 12460467 10.1111/j.1349-7006.2002.tb01231.x PMC5926901

[bib14] Harwerth IM, Wels W, Schlegel J, Müller M, Hynes NE (1993) Monoclonal antibodies directed to the erbB2 receptor inhibit *in vivo* tumour cell growth. Br J Cancer 68(6): 1140–1145 7903153 10.1038/bjc.1993.494 PMC1968669

[bib15] Hiesiger EM, Hayes R, Pierz DM, Budzilovich GN (1993) Prognostic relevance of epidermal growth factor receptor and neu/erbB2 expression in glioblastomas. J Neurooncol 16(2): 93–104 7507162 10.1007/BF01324695

[bib16] Koka V, Potti A, Forseen SE, Pervez H, Fraiman GN, Koch M, Levitt R (2003) Role of HER2/neu overexpression and clinical determinants of early mortality in glioblastoma multiforme. Am J Clin Oncol 26(4): 332–335 12902879 10.1097/01.COC.0000020922.66984.E7

[bib17] Kono K, Takahashi A, Ichihara F, Sugai H, Fujii H, Matsumoto Y (2002) Impaired antibody-dependent cellular cytotoxicity mediated by Herceptin in patients with gastric cancer. Cancer Res 6(20): 5813–5817 12384543

[bib18] Kristt DA, Yarden Y (1996) Differences between phosphotyrosine accumulation and neu/erbB-2 receptor expression in astrocytic proliferative processes: Implication for glial oncogenesis. Cancer 78(6): 1272–1283 8826951 10.1002/(SICI)1097-0142(19960915)78:6<1272::AID-CNCR16>3.0.CO;2-Y

[bib19] Lewis GD, Figari I, Fendly B, Wong WL, Carter P, Gorman C, Shepard HM (1993) Differential responses of human receptor cell lines to anti185 HER2 monoclonal antibody. Cancer Immunol Immunother 37(4): 255–263 8102322 10.1007/BF01518520 PMC11038979

[bib20] Mineo JF, Quintin-Roue I, Lucas B, Buburuzan V, Besson G (2002) Les glioblastomes: Etude clinique et recherche de facteurs pronostiques. Neurochirurgie 48(6): 500–509 12595806

[bib21] Niculescu P, Rus HG, Retegan M, Vlaicu R (1992) Persistent complement activation on tumour cells in breast cancer. Am J Pathol 140: 1039–1043 1374587 PMC1886512

[bib22] Pegram M, Hsu S, Lewis G, Pietras R, Beryt M, Sliwkowski M, Coombs D, Baly D, Kabbinavar F, Slamon D (1999) Inhibitory effects of combination of Her-2/neu antibody and chemotherapeutic agents used for treatment of human breast cancers. Oncogene 18(13): 2241–2251 10327070 10.1038/sj.onc.1202526

[bib23] Peress NS, Fleit HB, Perillo E, Kuljis R, Pezzullo C (1993) Identification of the FC gamma RI, II and III on normal human brain ramified microglia and on microglia in senile plaques in Alzheimer's disease. J Neuroimmunol 48(1): 71–79 8227309 10.1016/0165-5728(93)90060-c

[bib24] Pietras RJ, Pegram MD, Finn RS, Maneval DA, Slamon D (1998) Remission of human cancer xenografts on therapy with humanized monoclonal antibody to HER2 receptor and DNA reactive drugs. Oncogene 17(17): 2235–2249 9811454 10.1038/sj.onc.1202132

[bib25] Press MF, Cordon-Cardo C, Slamon DJ (1990) Expression of the HER2/neu proto-oncogene in normal adult and fetal tissues. Oncogene 5(7): 953–962 1973830

[bib26] Puzzili F, Ruggeri A, Mastronardi L (1998) Long term survival in cerebral glioblastoma. Tumori 84: 69–74 9619719 10.1177/030089169808400115

[bib27] Rainov N, Dobberstein K, Bahn H, Holzhausen H, Lautenschlager C, Heidecke V, Burkert W (1997) Prognosis factors in malignant glioma: Influence of the overexpression of oncogene and tumor suppressor gene products on survival. J Neurooncol 35(1): 13–28 9266437 10.1023/a:1005841520514

[bib28] Sliwkowski M, Lofgren J, Lewis G, Hostaling T, Fendly B, Fox J (1999) Nonclinical studies addressing the mechanism of action of trastuzumab. Semin Oncol 26(4, Suppl 12): 60–70 10482195

[bib29] Spiridon CI, Ghetie MA, Uhr J, Marches R, Li JL, Shen GL, Vitetta ES (2002) Targeting multiple Her-2 epitopes with monoclonal antibodies result in improved antigrowth activity of a human breast cancer cell line *in vitro* and *in vivo*. Clin Cancer Res 8(6): 1720–1730 12060609

[bib30] Thomas CY, Chouinard M, Cox M, Parson S, Stalling-Mann M, Garcia R, Jove R, Wharen R (2003) Spontaneous activation and signaling by overexpressed epidermal growth factor receptors in glioblastoma cells. Int J Cancer 104(1): 19–27 12532415 10.1002/ijc.10880

[bib31] Vajkoczy P, Schilling L, Ullrich A, Schmiedek P, Menger M (1998) Characterization of angiogenesis and microcirculation of high-grade glioma: an intravital multifluorescence microscopic approach in the athymic nude mouse. J Cereb Blood Flow Metab 18(5): 510–520 9591843 10.1097/00004647-199805000-00006

[bib32] Vedeler C, Ulvestad E, Grundt I, Conti G, Nyland H, Matre R, Pleasure D (1994) Fc receptor for igG on rat microglia. J Neuroimmunol 49(1): 19–24 8294556 10.1016/0165-5728(94)90176-7

[bib33] Westphal M, Meima L, Szonyi E, Lofgren L, Meissner H, Hamel W, Nikolics K, Sliwkowski M (1997) Heregulins and the Erb-2/3/4 receptors in gliomas. J Neurooncol 35: 335–346 9440030 10.1023/a:1005837122181

